# Whole genome SNP genotype piecemeal imputation

**DOI:** 10.1186/s12859-015-0770-2

**Published:** 2015-10-23

**Authors:** Yining Wang, Tim Wylie, Paul Stothard, Guohui Lin

**Affiliations:** 1 Department of Computing Science, University of Alberta, Edmonton, Alberta T6G 2E8 Canada; 2 Currently with Department of Computer Science, University of Texas – Rio Grande Valley, Edinburg, 78539 Texas USA; 3 Department of Agricultural, Food, and Nutritional Science, University of Alberta, Edmonton, T6G 2C8 Alberta Canada

## Abstract

**Background:**

Despite ongoing reductions in the cost of sequencing technologies, whole genome SNP genotype imputation is often used as an alternative for obtaining abundant SNP genotypes for genome wide association studies. Several existing genotype imputation methods can be efficient for this purpose, while achieving various levels of imputation accuracy. Recent empirical results have shown that the two-step imputation may improve accuracy by imputing the low density genotyped study animals to a medium density array first and then to the target density. We are interested in building a series of staircase arrays that lead the low density array to the high density array or even the whole genome, such that genotype imputation along these staircases can achieve the highest accuracy.

**Results:**

For genotype imputation from a lower density to a higher density, we first show how to select untyped SNPs to construct a medium density array. Subsequently, we determine for each selected SNP those untyped SNPs to be imputed in the add-one two-step imputation, and lastly how the clusters of imputed genotype are pieced together as the final imputation result. We design extensive empirical experiments using several hundred sequenced and genotyped animals to demonstrate that our novel two-step piecemeal imputation always achieves an improvement compared to the one-step imputation by the state-of-the-art methods Beagle and FImpute. Using the two-step piecemeal imputation, we present some preliminary success on whole genome SNP genotype imputation for genotyped animals via a series of staircase arrays.

**Conclusions:**

From a low SNP density to the whole genome, intermediate pseudo-arrays can be computationally constructed by selecting the most informative SNPs for untyped SNP genotype imputation. Such pseudo-array staircases are able to impute more accurately than the classic one-step imputation.

## Background

*Genome-wide association studies* (GWAS) are processes of genetic fine-mapping that find whether common genetic variants are associated with a trait of interest [1]. These common genetic variants are expected to be abundant and well distributed across the whole genome. One of the most commonly used variant types is the *single nucleotide polymorphism* (SNP) — a site in the genome at which different individuals display different nucleotides. In this paper we consider biallelic SNPs in the cattle genome. The 1000 Bull Genomes Project (www.1000bullgenomes.com) has identified more than 28 million single nucleotide variations in cattle. The number of biallelic SNPs with significant minor allele frequency is expected to be around 26.7 million (*we use this number throughout the paper*), and they are almost evenly distributed across the cattle genome. If we were given the genotype for all 26,700,000 SNPs for all studied individuals, a GWAS would simply be a pure association study. In reality, it is still too expensive to sequence the whole genome of every individual in the study; for most cattle GWAS, instead only a small fraction of the 26,700,000 SNPs are genotyped using commercial arrays. Two series of general purpose commercial gene chips for SNP genotyping in cattle are the Illumina (www.illumina.com) series and the Affymetrix (www.affymetrix.com) series. In our cattle genomics projects, besides sequencing 30 or so individuals per breed, we have genotyped thousands of animals using the Illumina BovineHD BeadChip (Illumina 777 K) that contains more than 777,000 SNPs and the Axiom Genome-Wide BOS 1 Array (Affymetrix 660 K) that contains more than 648,000 SNPs spanning the entire bovine genome. In addition, we have certain low-density and medium-density genotype datasets obtained using the Illumina BovineLD BeadChip (Illumina 6 K) and the Illumina BovineSNP50 BeadChip (Illumina 50 K), respectively.

The project goal discussed in this paper is to use the sequenced animals as *references* to impute the whole genomes (that is, the 26,700,000 SNP genotype values) for the animals genotyped at various lower density levels, a scenario we designate as the *whole genome SNP genotype imputation*. For example, the Illumina 6 K chip provides genotypes for around 6000 SNPs across the 29 autosomes, and the animals genotyped with the Illumina 6 K chip will have the remaining 26,694,000 SNPs imputed up from the 6000 SNP genotype, based on the sequence-derived SNP genotypes of the reference sequenced animals. The subsequent GWAS may be done on either the 6000 (i.e. 0.2 %) genotyped SNPs, or can also be done on the imputed genotype including the other 26,694,000 (i.e. 100 %) SNPs.

Note that a typical GWAS genotype dataset that contains thousands of SNPs and hundreds to thousands of individuals is apt to contain missing genotype data. Besides discarding the concerned SNPs from GWAS or perhaps repeating genotyping experiments, computationally inferring the missing data has long been proposed as an alternative, at minimal labor and cost [2–5]. In other words, SNP genotype imputation is not a novel topic, but has received extensive research attention since the start of genotyping arrays — see reviews and surveys [6–11]. Nevertheless, in this paper, our imputation target is not the small percentage of missing genotype for SNPs on the gene chips, but the untyped SNPs. Such an objective is becoming increasingly relevant, thanks to ongoing efforts to make sequenced animals available as references.

All existing genotype imputation methods are based upon the *coalescent* theory, which states that a short (i.e. over a short chromosomal region) haplotype allele is expected to be shared by many individuals due to *identical-by-descent* inheritance. Computationally, these methods can be classified into regression and clustering [6, 12–15], hidden Markov models (HMMs) and expectation maximization (EM) algorithms [7]. Among others [2, 4, 16–19], the most promising and applicable methods include fastPHASE [5], MaCH [20], and Impute [21, 22] which are based on Li and Stephens’ “product of approximate conditionals” framework [23], Beagle [24] which is based on Brownings’ “localized haplotype clustering” model [25], Mendel-Impute [26] using “low rank based matrix completion”, and FImpute [27] which is based on “long-range phasing” [28]. All these methods can potentially be adopted for our purpose to impute the genotype values for the large number of untyped SNPs, with varying speed and accuracy.

More specifically in our whole genome SNP genotype imputation projects, the higher density chip is meant to be the whole genome sequence data, consisting of all 26.7 million SNPs; the lower density chip can be either of the Illumina 6 K, 50 K, 777 K, or the Affymetrix 660 K. Running an imputation method one time to directly impute the whole genome sequences is referred to as the *one-step* imputation, which is *not* the computational problem we try to address in this paper. Recently, several studies showed evidence that within bovine genomics, the *two-step* imputation is generally more accurate than the one-step imputation, where the lower density genotyped animals are first imputed to a medium density SNP set and then further imputed to the higher density [29–31]. For instance, Larmer et al. showed that for Beagle, FImpute, and Impute v2 [22], the two-step imputation from 6 to 50 K then to 777 K achieves higher accuracies than the one-step imputation from 6 K directly to 777 K [30].

In our preliminary studies, we have conducted the so called *add-one two-step imputation* experiments, in which the median density reference panel contains only one extra SNP than the low density SNP panel. While rotating this extra SNP from the pool of markers in the high density panel, we observed that a portion of them can individually boost the imputation accuracy in the add-one two-step experiment compared to the one-step direct imputation. Inspired by this observation, we are asking the natural question whether building up a staircase of pseudo arrays in between the lower density SNP set and the designated higher density SNP set would give the best imputation result. In this paper, we attempt to answer this question by first presenting a novel two-step piecemeal imputation framework, which essentially builds an intermediate *pseudo* array by mining the *hidden* relations between the lower and the higher density arrays. We remark that our pseudo array is not an actually manufactured gene chip, but an artificial one that is computationally derived from a learning procedure, which evaluates and selects some SNP markers based upon their add-one two-step imputation performance. Moreover, the pseudo-arrays are model-dependent, that is, different base imputation programs built upon different models could result in different selection of markers for our two-step piecemeal imputation. We show that by wrapping either Beagle or FImpute in our two-step piecemeal imputation framework, we are able to achieve higher genotype imputation accuracies. (*That is, our method will be based on the one-step imputation, and is hunting for improvement upon the corresponding one-step imputation from the data.*) Though we believe most effective imputation methods mentioned earlier can be adopted, the main reason we only go with Beagle and FImpute is their fast speed (i.e. efficiency). Based on the two-step piecemeal imputation, we demonstrate how staircase arrays can be built for whole genome SNP genotype imputation.

We briefly describe our two-step piecemeal imputation framework here (see Fig. 3 for a flow chart); more details are provided in the Methods section. By a *study sample*, we mean an animal that is genotyped at the lower density. The SNPs on this lower density chip form a set *T*. A *reference panel* consists of individuals genotyped at the higher density. All SNPs of *T* are assumed to be assayed in the higher density chip; the other SNPs on the higher density chip but not in *T* form the set *U* — they are the untyped SNPs in the study samples. For each marker *m*_*i*_∈*U*, an *add-one* two-step imputation from *T* to *T*∪{*m*_*i*_} to *T*∪*U* is conducted to evaluate its *potential* in imputing the other untyped markers. Next, markers that have similar potentials are clustered together, and correspondingly for each *marker cluster*, we determine which other markers can be imputed well, forming into a *target marker cluster*. We select one marker from every marker cluster, together with *T*, to form the pseudo intermediate array. The SNPs in a target marker cluster, called a *tract*, are imputed via the add-one two-step imputation where the selected marker is from the associated marker cluster. *Piecing* these tracts together gives the final imputation result.

## Results

We first evaluate our two-step piecemeal imputation method empirically, and make comparisons against the usual one-step imputation; we also compare our multi-step piecemeal imputation method against the usual two-/three-step imputations. In this empirical evaluation, we use Beagle (version 3.3.2) and FImpute (version 2.2) as the two base imputation programs respectively, which are fast and memory efficient enough to run them thousands of times on multiple large to huge datasets.

### Datasets

#### Sequenced animals

The Canadian Cattle Genome Project (http://www.genomecanada.ca) has contributed more than 350 animals to the 1000 Bull Genomes Project. From these projects we derived two datasets: a Holstein sequence collection containing 114 animals, and a Simmental sequence collection containing 82 animals. They were used for the piecemeal imputation method training through a cross validation process (i.e. partitioned into a subset of study samples and another subset of reference samples). They also served as reference samples in all the independent testing experiments.

#### Genotyped animals

From the Canadian Cattle Genome Project, we obtained Affymetrix 660 K genotypes for 390 Simmental animals. There are 23 of these 390 Simmental animals included in the set of sequenced animals. The genotyped animals that are not included in the set of sequenced animals are used as study samples in the independent testing experiments from a lower (than 660 K) density to impute their genotypes at the density 660 K. The 23 genotyped and sequenced animals are used as study samples in the independent testings from a lower density to impute their whole genome.

#### SNP sets

We used single chromosomes of small size (BTA 27) or medium size (BTA 14) in the development of the piecemeal imputation framework. BTA 27 was used for the Holstein data set and BTA 14 for the Simmental data set. The only challenge to deal with all 29 chromosomes is the need of a huge amount of disk storage, see Discussion.

The numbers of SNPs included in the Illumina 6 K, 50 K, 777 K and the Affymetrix 660 K are summarized in Table 1, where the second column contains their formal chip names that one can look up on the Illumina and Affymetrix websites.

**Table 1:** Description of the different SNP chips and the SNP subsets

SNP Chip	Chip Name	#SNPs
Illumina 6 K	Illumina BovineLD BeadChip	6,909
Illumina 50 K	Illumina BovineSNP50 BeadChip	54,001
Illumina 777 K	777 K BovineHD BeadChip	786,799
Affymetrix 660 K	Axiom Genome-Wide BOS 1 Array	648,875

On BTA 27, the 114 sequenced Holstein animals have genotype values for 529,674 SNPs. The Illumina 777 K chip contains 10,219 of them, among which 664 are included in the 50 K chip, and 119 of these 664 SNPs are included in the 6 K chip, as summarized in Table 2. On BTA 14, the 82 sequenced Simmental animals have genotype values for 933,833 SNPs. Table 2 shows that the Affymetrix 660 K chip contains 14,367 of these 933,833, among which 1618 are included in the 50 K chip, and a further 219 of these 1,618 SNPs are included in the 6 K chip.

**Table 2:** Description of the different SNP chips and the filtered SNP subsets used in the study

Chr	#Animals	#SNPs	HD	50 K	6 K
BTA 27	114	529,674	10,219	664	120
BTA 14	82	933,833	14,367	1,618	219

### Imputation results

#### 5-fold cross validation

We use 5-fold cross validation to empirically examine our piecemeal imputation method, also to construct (a.k.a. train) the staircase pseudo arrays to impute the genotyped animals to their whole genome. The cross validation results also suggest the possible levels of improvement compared with the one-step imputation.

**Fig. 1:**
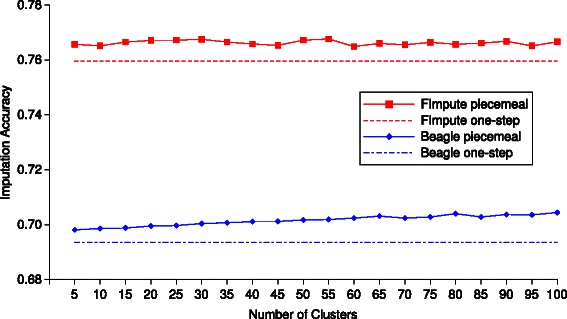
The Beagle/FImpute-based two-step piecemeal imputation accuracies against the number of SNP clusters

Table 3 contains the cross validation results on the Simmental datasets of 82 animals, where the lower density is either 6 or 50 K and the higher density refers to either 50 or 660 K (second column). The third and the fourth columns hold the one-step and piecemeal imputation accuracies (*a* *a* *c*_1_ and *a* *a* *c*_*π*_, respectively), and the improvement of piecemeal over one-step is shown in the fifth column. For the null hypothesis that the usual one-step accuracies and the two-step piecemeal imputation accuracies have equal mean accuracies, we conducted statistical significance testing. Using Beagle as the base program, the *p*-values for the three 5-fold cross validation experiments are 0.0215, 0.0005 and 0.0004, respectively, indicating that the improvements by the two-step piecemeal imputation are statistically significant; using FImpute, the corresponding *p*-values are 0.64, 0.49 and 0.61, suggesting statistically insignificant improvements. From 6 to 50 K, 5 to 100 marker clusters, in increments of 5, were examined and the best piecemeal imputation results are included in the table, while in Fig. 1 all of these accuracies are plotted (blue dots). From 6 or 50 K to 660 K, 100 to 1,000 marker clusters, in increments of 100, were examined.

**Table 3:** Accuracy comparisons between the two-step piecemeal and the classic one-step imputation on the Simmental datasets

		5-Fold cross validation					Independent testing		
BaseProgram	Imputation	*a* *c* *c*_1_	*a* *c* *c*_*π*_	+	#Clusters	#TClusters	*a* *c* *c*_1_	*a* *c* *c*_*π*_	+
	6 K →50 K	69.35 *%*	70.81 *%*	**1.46 %**	100	100	60.68 *%*	61.39 *%*	**0.71 %**
Beagle	6 K →660 K	72.37 *%*	74.92 *%*	**2.55 %**	800	800	66.00 *%*	67.76 *%*	**1.76 %**
	50 K →660 K	86.61 *%*	88.89 *%*	**2.28 %**	1000	1000	72.83 *%*	74.11 *%*	**1.29 %**
	6 K →50 K	75.95 *%*	76.70 *%*	**0.75 %**	55	55	61.87 *%*	62.16 *%*	**0.29 %**
FImpute	6 K →660 K	79.11 *%*	80.11 *%*	**1.00 %**	1000	1000	68.43 *%*	68.95 *%*	**0.52 %**
	50 K →660 K	90.31 *%*	90.74 *%*	**0.43 %**	1000	^1^999	77.11 *%*	77.33 *%*	**0.22 %**

Results are on the Simmental datasets for markers on chromosome 14. Columns 3–7 contain the 5-fold cross validation results on the 82 animals, with the selected markers and their associated target marker clusters. Independent testing results on the 367 animals are in columns 8–10, using the selected markers and their associated target marker clusters from the cross validation. ^1^In the independent testing from 50K to 660K, 8 markers of the Affymetrix 660K chip were filtered out due to their genotype disagreeing with the alternating alleles specified by sequencing, and consequently only 999 target marker clusters were used. The columns labelled with + show the improvements, in bold, of the piecemeal imputation over the one-step imputation

Analogous results on the Holstein datasets of 114 animals are shown in Table 4. Both tables show a 1.5– 3.0 *%* net accuracy improvement against Beagle, in all three cases (with statistical significance testing *p*-values 0.00369, 0.00003 and 0.00019, respectively) and a 0.5– 1.0 *%* net accuracy improvement against FImpute (with *p*-values 0.54, 0.38 and 0.31, respectively).

**Table 4:** Accuracy comparisons between the two-step piecemeal and the classic one-step imputation on the Holstein datasets

		5-Fold cross validation					Independent testing		
BaseProgram	Imputation	*a* *c* *c*_1_	*a* *c* *c*_*π*_	+	#Clusters	#TClusters	*a* *c* *c*_1_	*a* *c* *c*_*π*_	+
	6 K →50 K	86.98 *%*	89.81 *%*	**2.87 %**	95	^1^89	74.97 *%*	76.90 *%*	**1.94 %**
Beagle	6 K →777 K	82.35 *%*	85.27 *%*	**2.92 %**	1,000	^1^963	71.29 *%*	73.25 *%*	**1.96 %**
	50 K →777 K	93.09 *%*	95.16 *%*	**2.07 %**	1,000	^1^956	82.27 *%*	84.25 *%*	**1.97 %**
	6 K →50 K	91.11 *%*	91.64 *%*	**0.53 %**	95	^2^88	81.15 *%*	81.40 *%*	**0.25 %**
FImpute	6 K →777 K	89.22 *%*	90.14 *%*	**0.92 %**	1,000	^2^942	82.80 *%*	82.81 *%*	**0.02 %**
	50 K →777 K	95.25 *%*	95.61 *%*	**0.36 %**	800	^2^765	87.72 *%*	87.83 *%*	**0.11 %**

Results are on the Holstein datasets for markers on chromosome 27. Columns 3–7 contain the 5-fold cross validation results on 114 animals, with the selected markers and their associated target marker clusters. Independent testing results on the 8 animals are in columns 8–10, using the selected markers and their associated target marker clusters from the cross validation. In the independent testing, for ^1^Beagle 6, 37, and 44 target marker clusters are empty; for ^2^FImpute 7, 58, and 35 target marker clusters are empty. The columns labelled with + show the improvements, in bold, of the piecemeal imputation over the one-step imputation

#### Independent testing

Independent testing is to examine the quality of the selected markers and the defined pieces learned from the training step. The study samples used in the testing are not involved in the training step. The piecemeal imputation accuracies are again compared to the corresponding one-step imputation accuracies, respectively.

Columns 8–10 of Table 3 contain these independent testing results on the 367 genotyped Simmental animals where the lower density is either 6 or 50 K and the higher density refers to either 50 or 660 K (second column). The 8th and 9th columns hold the one-step and piecemeal imputation accuracies (*a* *a* *c*_1_ and *a* *a* *c*_*π*_), respectively. The improvement of the piecemeal over the one-step is shown in the tenth column. Note that for each imputation setting, the selected markers and the defined pieces are taken from the respective cross validation experiment. One exception is that there are 8 markers in the Affymetrix 660 K chip for which the two alleles (i.e. nucleotides) do not agree with the alternating alleles identified through genome sequencing; these 8 markers were excluded and one target marker cluster was discarded in the testing.

Analogous independent results on the 8 genotyped Holstein animals are shown in Table 4. Both tables show an accuracy improvement in all settings, though the improvement is about 40 *%* lower than the 5-fold cross validation.

#### Multi-step imputation: independent testing

With the selected markers and their associated target marker clusters from the training step, we experimented with the usual two-step imputation from 6 K to 50 K to 660 K on the 367 genotyped Simmental animals, and the four-step piecemeal imputation from 6 to 660 K. The four-step piecemeal imputation is a result of replacing each usual one-step imputation by a potentially promising two-step piecemeal imputation. The usual two-step imputation accuracy is denoted as *a* *c* *c*_2_; the four-step piecemeal imputation accuracy is still denoted as *a* *c* *c*_*π*_. Similar experiments were done on the 8 Holstein animals on BTA 27 genotyped using the 777 K chip.

For the 23 genotyped and sequenced Simmental animals, we experimented with the usual two-step imputation from 50 to 660 K to Sequence and the four-step piecemeal imputation from 50 K to Sequence, and the usual three-step imputation from 6 K to 50 K to 660 K to Sequence and the five-step piecemeal imputation from 6 K to Sequence. Here “Sequence” refers to all the 529,674 SNPs on BTA 14. The usual three-step imputation accuracy is denoted as *a* *c* *c*_3_; the five-step piecemeal imputation accuracy is denoted as *a* *c* *c*_*π*_. Note that since we do not have a 660 K to Sequence training step to select markers (because first the usual one-step imputation is very good leaving little room for further improvement and second the training phase requires storage beyond our capacity), the last step in the five-step piecemeal imputation is a direct one-step imputation.

All these usual two/three-step imputation accuracies and the corresponding four/five-step piecemeal imputation accuracies are summarized in Table 5, where there is accuracy improvement in all settings. We note that these 23 animals were used in the training step, and thus the results reported here could be slightly biased.

**Table 5:** Accuracy comparisons between the multi-step piecemeal and the usual two/three-step imputation

BaseProgram	Imputation	*a* *c* *c*_1_	*a* *c* *c*_2_	*a* *c* *c*_3_	*a* *c* *c*_*π*_	+
Beagle	8 Holstein BTA 27	71.29 *%*	74.25 *%*		74.43 *%*	**0.18 %**
FImpute	6 K →50 K →777 K	82.80 *%*	82.74 *%*		82.92 *%*	**0.18 %**
Beagle	367 Simmental BTA 14	66.00 *%*	65.51 *%*		66.59 *%*	**1.08 %**
FImpute	6 K →50 K →660 K	68.43 *%*	68.54 *%*		68.56 *%*	**0.02 %**
Beagle	23 Simmental BTA 14	84.91 *%*	89.88 *%*		90.17 *%*	**0.29 %**
FImpute	50 K →660 K →Sequence	87.95 *%*	90.47 *%*		90.50 *%*	**0.03 %**
Beagle	23 Simmental BTA 14	81.19 *%*		83.94 *%*	86.26 *%*	**2.32 %**
FImpute	6 K →50 K →660K →Sequence	82.23 *%*		84.58 *%*	84.67 *%*	**0.09 %**

Results are on the Holstein datasets for markers on chromosome 27 and for the Simmental datasets for markers on chromosome 14, respectively. 8 Holstein and 367 Simmental genotyped animals are used in the two-step independent testing (6 K →50 K →HD), with results in columns 4, 6 and 7. The piecemeal imputation uses the selected markers and their associated target marker clusters from the training step. Additional 23 Simmental sequenced and genotyped animals are used in the two/three-step imputation to Sequence (50K →660K →Sequence, 6K →50K →660K →Sequence). All one-step imputation accuracies are included in column 3. The last column labelled with + show the improvements, in bold, of the piecemeal imputation over the two- or three-step imputation

## Discussion

### Rationale behind the two-step piecemeal imputation

Several recent studies in cattle have shown that two-step imputation can be more accurate than the classic one-step imputation [29–31]. Also in our preliminary study, we observed that some markers in the add-one two-step imputation experiments are able to boost the overall accuracy. These results have led us to put efforts into finding a set of markers that would perform the best in the subsequent two-step imputation. However, such an optimal set of markers is not assayed in any existing chips, nor easy to obtain in reasonable computational time. Besides the selection scheme in our piecemeal imputation framework, we also tried several other approaches including *sequential forward selection*, which did not result in any significant improvement. We thus proposed an alternative to partition the higher density SNP set into multiple pieces, which are learned through the add-one two-step imputation experiments. Each piece is then imputed by the corresponding add-one two-step imputation experiment. This procedure laid the foundation for our two-step piecemeal imputation strategy. Nevertheless, our partition scheme is not necessarily optimal, as we adopted the *k*-means only because it outperformed other clustering methods slightly. In addition, we also experimented with the *linkage-disequilibrium* (LD) blocks produced by Haploview [32] for finding closely linked markers, but again the increase in accuracy was insignificant and the results are often inferior to those of the our marker selection scheme (detailed results not shown).

### Marker clusters and their effects

From our 5-fold cross validation results, it seems as though the number of marker clusters does not affect the final piecemeal imputation accuracy much. For example, for genotype imputation from 6 to 50 K on the Simmental dataset, the piecemeal imputation accuracies of all the 20 different clustering results are plotted in Fig. 1, where the dashed blue/red lines are the Beagle/FImpute one-step imputation accuracies, and the solid dots represent the two-step piecemeal imputation accuracies. Despite FImpute performing better than Beagle, the connected dots for both FImpute and Beagle do not vary much with different numbers of clusters. A simple guideline would be to have an average cluster size of 10–100.

**Fig. 2:**
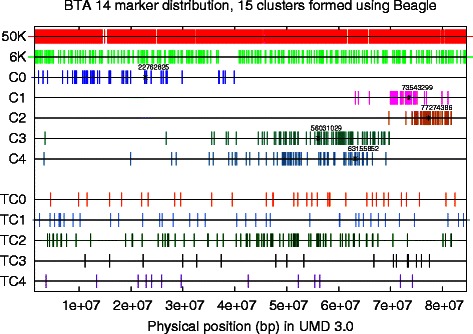
Untyped SNP genotype piecemeal imputation. Both the SNP set *T* of a lower density 6 K chip and the SNP set *T*∪*U* of a higher density 50 K chip are shown, using their physical loci on BTA 14. The second to the seventh lines plot the SNPs in the first five clusters, by the *k*-means algorithm (*k*=15) on the marker feature vectors generated by the add-one two-step imputation using Beagle. The starred markers are the selected markers, one per cluster, and the associated target marker clusters are shown in the last five lines in the figure

We also look into the content of a marker cluster. For example, when *k*=15, the first five of the 15 marker clusters are plotted in Fig. 2, where the *x*-axis represents the physical locus. It is interesting to see that the markers of a cluster are not necessarily close to each other, though they have very similar imputation potentials. The LD between pairs of these markers, by Haploview, are insignificant.

### Imputation result sensitivity to the selected markers

The imputed genotype for the study samples at a selected marker *m*_*i*_ is used in the second step, of the two-step piecemeal imputation, to impute the other untyped markers of *U*−{*m*_*i*_}. Comparing the add-one two-step imputation result to the usual one-step imputation, we have seen subtle changes at many untyped markers of *U* for different selected markers. Indeed, some of them exhibit a gain in accuracy whereas some have a loss in accuracy and yet others are unaffected. This has led us to use the overall gain in accuracy to measure the *imputation potential* of a selected marker.

By setting up a feature vector for a candidate marker to keep a record of the accuracy gains and losses at each untyped marker, we observed from our preliminary two-step imputation experiments (results not shown) that the candidate markers fall into three categories when used for creation of the pseudo-array: 1) those that yield an accuracy gain over the usual one-step imputation; 2) those that yield a net zero gain; 3) those that yield an accuracy loss from the usual one-step imputation. Through clustering these feature vectors, the impact of selecting different markers from a cluster is expected to be reduced to the minimum, as evidenced by our preliminary experiments (we did not re-examine this issue in all the experiments reported here).

### Target marker clusters

For all the markers of a marker cluster, those other untyped markers that can be similarly well imputed in the add-one two-step imputation are the target markers associated with the marker cluster. We have looked into the content of such a target marker cluster. Similar to a marker cluster, it is interesting to see that the markers of a target cluster are not necessarily physically close to each other, nor are the LD between pairs of these markers by Haploview significant.

It is also interesting to observe that some target marker clusters are overlapping. Note that target clusters are formed after the marker clusters are determined, that is, in terms of the feature vectors, the marker clusters are formed using the whole vectors, but the target marker clusters are formed by using only the vector entries corresponding to the makers in a marker cluster. Therefore, such a phenomenon of an untyped marker being imputed with high accuracies by several selected markers can be explained.

### Other clustering methods

The main reason for marker clustering is to avoid selecting redundant markers to form the intermediate pseudo array, here redundant means the similar potential in imputing the genotype for other SNPs. We had experimented with Haploview to construct the LD blocks for this purpose, which did not result in any conclusive accuracy increase (detailed results not shown). Other popular feature selection methods in machine learning, such as SFS and SBS, were also tested. Based on the feature vectors, we tried clustering methods other than *k*-means, with results not better than *k*-means. Thus we go with *k*-means in the final piecemeal imputation framework.

As discussed in the last paragraph, forming the marker clusters and the associated target marker clusters is more like a *bi-clustering* task, and it would be worthwhile to try some good bi-clustering algorithms. Coming back to the LD-based marker selection, though multiple experiments with different thresholds in Haploview did not give good results, we realize that such an approach avoids the add-one two-step imputation experiments in the training phase, and it can be substantially faster. This suggests the need for better LD block estimation/prediction by SNP genotype values.

### Cattle genomic distance

In our current empirical experiments, we used the population-based option in our base programs. The underlying assumption for such an option is that individuals are unrelated. On the other hand, related animals can certainly bias towards the correct genotype. Therefore, if one would be able to define a degree of relatedness between two individuals based on their SNP genotype, then using only closely related sequenced animals to a study animal as references may potentially lead to more accurate genotype imputation.

Animals from different breeds are deemed more distantly related than the same breed animals. We therefore separated the datasets by breeds. In fact, earlier research suggests cattle whole genome SNP genotype imputation should be done breed by breed [30], which is also confirmed by our preliminary testing that inter-breed imputation has slightly lower performance (detailed results not shown).

### Computational time

The running time of the two-step piecemeal imputation depends on the number of study animals, the number of reference animals, and the higher SNP density. Our experiments were mostly done using the high-performance computing facilities, and we were able to run them in parallel. The same as any other machine learning tasks, the most time-consuming stage in the two-step piece imputation is the training phase, when the add-one two-step experiments are conducted to select the good potential markers. The independent testing, and the real imputation tasks, can be finished much quicker. It is important to point out that in all our experiments reported here, running time was never an issue as it was in hours per run, but the major challenge is the need for a huge disk storage (more than 84 TB) when we were performing whole-genome SNP genotype imputation; and again this was happening in the training phase. We used more than 3TB for storing all the intermediate data, such as the feature vectors, for the two datasets used in this paper. The imputed SNP genotype values are expected to be useful in the downstream data analysis, such as GWAS, and thus the increased computation burden in the piecemeal imputation framework becomes worthy.

## Methods

**Fig. 3:**
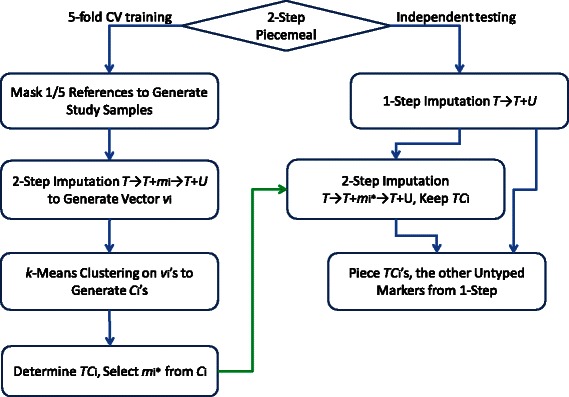
A flow chart of the two-step piecemeal imputation framework, including both the training phase through a 5-fold cross validation and the independent testing. In the chart, *T* is the set of markers in the lower density chip and *T*∪*U* is the set of markers in the higher density chip; *m*_*i*_ is a marker of *U*; *S* is the set of study samples genotyped on *T* and *R* is the set of references genotyped on *T*∪*U*. The goal is to impute the genotype for markers of *U* for the study samples

A flow chart of the two-step imputation process is depicted in Fig. 3, with the training process through the 5-fold cross validation in the left and the independent testing in the right. For ease of presentation, we use the Illumina 6 K gene chip to represent the lower density chip and the Illumina 50 K gene chip to represent the higher density one. The study samples are genotyped on the 6 K SNP set *T*, and the reference samples are genotyped on the 50 K SNP set *T*∪*U*. The goal is to impute the genotype values on *U* for the study samples. The top two lines in Fig. 2 plot *T*∪*U* and *T*, respectively, using their physical loci on the first half of chromosome 14 (BTA 14).

### One-step imputation

We first present the training process. Let Beagle be our base imputation algorithm. Denote the set of study samples as ${\mathcal S}$ and the set of reference samples as ${\mathcal R}$. The genotype dataset is thus denoted as $({\mathcal S} \cup {\mathcal R}, T \cup U)$. First, Beagle is used to impute the genotype of the study samples on the untyped SNPs of *U*, by simply running on the dataset $({\mathcal S} \cup {\mathcal R}, T \cup U)$. The achieved accuracy in this one-step imputation is denoted as *a* *c* *c*_1_. In our simulation experiments, the genotype of the study samples on the SNPs of *U* are masked; the imputed genotype is then compared against the true genotype for calculating the imputation accuracy.

### Marker feature vector

Our goal of training is to select a relatively small number of SNPs from *U*, denoted as *M*, and append them to set *T* to create an intermediate pseudo array, such that the subsequent two-step imputation from *T* to *T*∪*M* and then to *T*∪*U* yields a higher imputation accuracy. In this step, we will evaluate the *potential* for each SNP of *U* in the add-one two-step imputation.

Let *m*_*i*_∈*U* be a candidate marker (*i*=1,2,…,|*U*|), and tentatively set the intermediate pseudo array to contain *T* and *m*_*i*_ only, that is *T*∪{*m*_*i*_}. Applying the base imputation algorithm (Beagle in our case), we do the add-one two-step imputation from *T* to *T*∪{*m*_*i*_} and then from *T*∪{*m*_*i*_} to *T*∪*U*. At the end of the two-step process, we calculate the imputation accuracy for each marker *m*_*j*_ of *U* across all study samples, denoted as *a*_*ij*_. Let *a*_*ii*_ denote the one-step imputation accuracy from *T* to *T*∪{*m*_*i*_}. The vector *v*_*i*_=〈*a*_*i*1_,*a*_*i*2_,…,*a*_*i*|*U*|_〉, is the *feature vector* for marker *m*_*i*_.

### Marker clustering and target marker cluster

Intuitively, two markers of similar feature vectors have about the same performance in the two-step imputation, in terms of imputing the other untyped markers; and thus it is sufficient to include only one of them. Therefore, we apply the *k*-means clustering algorithm to cluster the markers of *U* represented by their feature vectors, where *k* is the pre-set number of clusters which can be empirically determined, for example, by the Davies-Bouldin index [33]. In our experiments, for genotype imputation from the Illumina 6 to 50 K using Beagle, we examined 5 to 100 clusters with an increment of 5 in the cross validation. Let *C*_1_,*C*_2_,…,*C*_*k*_ denote the *k* clusters of SNPs of *U* by *k*-means.

For each cluster *C*_*i*_, if marker *m*_*j*_∈*U* is consistently imputed well with average imputation accuracy higher than or equal to the one-step imputation accuracy *a* *c* *c*_1_, then *m*_*j*_ is a target marker for the cluster *C*_*i*_. The set of all target markers for cluster *C*_*i*_ form the target marker cluster *T* *C*_*i*_ for the cluster *C*_*i*_.

If the target marker cluster *T* *C*_*i*_ is empty, then no marker of *C*_*i*_ would be selected to form the pseudo array; otherwise, define the contribution of a marker *m*_*j*_ of *C*_*i*_ as the add-one two-step imputation accuracy from *T*∪{*m*_*j*_} to *T*∪*U*, with the imputation accuracy calculated over only markers of *T* *C*_*i*_. The top contribution marker of *C*_*i*_, denoted as $\phantom {\dot {i}\!}m_{i^{*}}$, is selected into *M*, and it is for imputing the genotype of SNPs of *T* *C*_*i*_ only. We call this target marker cluster *T* *C*_*i*_ one *piece* of the imputation.

### Experimental setup in the two-step piecemeal imputation

We have two sets of sequenced animals (Holstein and Simmental) to be used as references. There are also much larger numbers of animals that are genotyped by various Illumina and Affymetrix gene chips, which are the study samples. The project goal is to impute the study animals to their whole sequences. When running the base programs in our experiments, we followed the default settings of the population-based genotype imputation for both Beagle and FImpute. For Beagle, we set the -Xmx parameter to 4GB for memory management without the low memory option for better performance.

#### Training through cross validation

Recall that we have four levels of SNP density, 6 K, 50 K, HD and Sequence, where HD can be either the Illumina 777 K or the Affymetrix 660K. For each genotype imputation experiment from a lower density to a higher density, we want to select some untyped SNPs to form the pseudo array and partition the other untyped markers into pieces accordingly. We do this training via a 5-fold cross validation. The quality of these selected markers and their associated pieces is examined via independent testing.

We use an example genotype imputation experiment for the Simmental group from 6 K to 50 K to explain the procedure. First, we derive the 50 K genotype for all sequenced Simmental animals from their sequence data. Next, these animals are randomly partitioned into five folds of approximately equal size, with the consideration of their country of origins. Four folds of the animals are used as the references, ${\mathcal R}$, while the last fold is held as study samples, ${\mathcal S}$, for which the genotype of SNPs outside of 6 K are masked to mimic the untyped SNPs *U*. The five-fold cross validation scheme rotates each one of the five folds as the study fold.

On the dataset $({\mathcal R} \cup {\mathcal S}, T \cup U)$, using Beagle we do the one-step imputation to obtain the imputation accuracy *a* *c* *c*_1_; we also do the add-one two-step imputation to obtain the feature vector for each untyped marker of *U*. The average of the five feature vectors from the five-fold cross validation defines the final feature vector for each untyped marker. With these vectors, the *k*-means algorithm is run to cluster the untyped markers into *C*_1_,*C*_2_,…,*C*_*k*_ (which form a partition of *U*). Subsequently, we determine the target marker cluster *T* *C*_*i*_ for each cluster *C*_*i*_ (all these target marker clusters form another distinct partition of *U*), and select the markers $\phantom {\dot {i}\!}M = \{m_{1^{*}}, m_{2^{*}}, \ldots, m_{k^{*}}\}$ for piecemeal imputation. For each study fold ${\mathcal S}$, the study samples are then piecemeal imputed to fill the genotype for the untyped markers *U*: when an untyped marker does not belong to any target marker cluster, it is imputed in the one-step, otherwise, it is imputed in multiple pieces through a majority vote. The imputed genotype values are compared against the ground truth, which were masked before the imputation, to calculate the piecemeal imputation accuracy as the percentage of correctly imputed genotype. The final piecemeal imputation accuracy from the five-fold cross validation is the average over all five folds, and is denoted as *a* *c* *c*_*π*_.

#### Independent testing

In these experiments, the sequenced animals from the Canadian Cattle Genome Project and the 1000 Bull Genomes Project are used as references. The study samples are the animals that are genotyped by various Illumina and Affymetrix gene chips.

We use the example genotype imputation experiment for the Simmentals from 6 to 50 K to explain the procedure. First, we derive the 50 K genotype for all sequenced Simmental animals from their sequence data; we also derive the 6 K genotype for the genotyped Simmental animals from their genotype data (they are genotyped at density 50 K or higher). Second, from the above 5-fold cross validation process, we have identified a set of markers $\phantom {\dot {i}\!}M = \{m_{1^{*}}, m_{2^{*}}, \ldots, m_{k^{*}}\}$ and determined their associated target marker clusters {*T* *C*_1_,*T* *C*_2_,…, *T* *C*_*k*_}, respectively. Using these selected markers and their defined pieces, our piecemeal imputation imputes the genotype for the study samples for the selected markers $\phantom {\dot {i}\!}M = \{m_{1^{*}}, m_{2^{*}}, \ldots, m_{k^{*}}\}$ first, and later uses the imputed data to further impute the genotype for the study samples at the other untyped SNPs. At the end, the imputed genotype for the study samples at all the untyped SNPs, either imputed in the first step or imputed in the second step, is compared against the ground truth to calculate the *independent testing piecemeal imputation accuracy* *a* *c* *c*_*π*_ as the percentage of correctly imputed genotype.

## Conclusions

In this study, we presented a novel two-step SNP genotype imputation strategy called piecemeal genotype imputation, which essentially inserts a pseudo intermediate density pseudo array in between the lower density chip and the target higher density chip. Using the two-step piecemeal imputation, we showed how a stair-case of intermediate SNP arrays can be built for the whole genome SNP genotype imputation. We applied this method to chromosomes 14 and 27 of cattle SNPs identified by whole genome sequencing, by carrying out multiple experiments using various density levels of bovine SNP chips, up to the sequence level. The results show preliminary success of our multi-step piecemeal imputation with an accuracy improvement compared to the classic one-step imputation by the state-of-the-art methods Beagle and FImpute.
